# Internet Addiction and Depressive Symptoms in University Students: Latent Profiles, Network Structure, and Symptomatic Pathways to Suicide Risk

**DOI:** 10.1155/da/4591408

**Published:** 2025-07-12

**Authors:** Yuan Li, Jing Shi, Biru Luo, Anqi Xiong, Siqi Xiong, Jing Wang, Shujuan Liao

**Affiliations:** ^1^Department of Nursing, West China Second University Hospital, Sichuan University/West China School of Nursing, Sichuan University, Chengdu 610041, China; ^2^Key Laboratory of Birth Defects and Related Diseases of Women and Children, Ministry of Education, Sichuan University, Chengdu 610041, China; ^3^Department of Neonatology, West China Second University Hospital, Sichuan University, Chengdu 610041, China; ^4^Department of Education, Sichuan Province, Chengdu 610041, Sichuan, China; ^5^West China School of Nursing, Sichuan University, Chengdu 610041, China; ^6^School of Nursing, Chengdu Medical College, Chengdu 610083, China; ^7^School of Nursing, Ya'an Polytechnic College, Ya'an 625000, China

**Keywords:** depressive symptoms, internet addiction, latent profile analysis, network analysis, suicide risk, university students

## Abstract

**Background:** Internet addiction and depression frequently co-occur among university students, resulting in amplified functional deterioration and treatment resistance. Despite established bidirectional relationships, existing research has predominantly examined linear associations and treated these conditions as single global constructs. This study integrated person-centered and network-based approaches to identify distinct symptom profiles of Internet addiction and depressive symptoms, examine sociodemographic predictors of profile membership, and uncover interconnected symptom networks within high-risk populations among Chinese university students.

**Methods:** A multicenter cross-sectional study was conducted from April to July 2024. Data were collected through a web-based survey incorporating validated instruments for Internet addiction, depression, and suicide risk assessment. Latent profile analysis was employed to identify distinct symptom profiles, followed by multivariate logistic regression to examine sociodemographic predictors. Network analysis was performed within the high-risk profile to unveil symptom interactions, central symptoms, bridge symptoms, and symptomatic pathways to suicide risk.

**Results:** Among 30,992 participants, latent profile analysis identified three distinct groups: Healthy profile (59.31%), at-risk profile (35.06%), and comorbidity profile (5.63%). Students who were female, ethnic minorities, in higher grade levels, and had prolonged Internet use showed increased risks of problematic profiles. Conversely, enrollment in bachelor's programs, science and medical majors, higher household income, and regular physical activity demonstrated protective effects. Network analysis revealed Internet preoccupation and fatigue as central symptoms, identified key bridge symptoms (e.g., offline negative affect, difficulty concentrating) linking the symptom clusters, and highlighted Internet withdrawal symptoms and depressed mood as critical pathways to suicide risk within the comorbidity profile.

**Conclusion:** This study identified distinct profiles of Internet addiction and depression comorbidity, with specific sociodemographic and lifestyle predictors informing targeted screening strategies. Network analysis revealed central symptoms and specific bridge symptoms connecting the conditions, while also identifying critical pathways to suicide risk in the Comorbidity profile, providing empirical evidence for developing precise and effective interventions.

## 1. Introduction

Internet use has become ubiquitous among university students, with recent global estimates indicating that over 95% of college-aged individuals are regular Internet users [1]. While the Internet serves as an essential tool for academic and social purposes, the risks of Internet overuse and potential addiction have emerged as significant mental health concerns [2]. Internet addiction, defined as online-related compulsive behavior reflecting an inability to control Internet use [3], affects adolescents and adults worldwide with prevalence rates ranging from 0.5% to 40.0% (pooled estimate: 7.02%) [4], and precipitates various psychiatric disorders, particularly depression [5, 6]. Depression represents another increasingly prevalent psychological challenge among university students, with meta-analyses indicating that approximately one-third experience clinically significant depressive symptoms [7]. These psychological difficulties substantially impact academic performance, social functioning, and overall well-being, manifesting in decreased academic achievement, elevated dropout rates, and even increased suicide risk [6–11].

The co-occurrence of Internet addiction and depressive symptoms frequently results in amplified functional deterioration and treatment resistance; nevertheless, this comorbidity is prevalent given their overlapping neurobiological and behavioral manifestations [6, 12]. Epidemiological evidence demonstrates a bidirectional relationship between these conditions: individuals with Internet addiction exhibit heightened vulnerability to depressive symptoms compared to their nonaddicted peers, while depressed individuals show increased susceptibility to developing Internet addiction relative to healthy controls [13, 14]. Although this bidirectional association has been well documented, with Internet addiction exacerbating depressive symptoms through social isolation and disrupted sleep–wake patterns, and depression driving excessive Internet use as a maladaptive coping mechanism, existing research has predominantly focused on linear associations between symptom severity, treating these conditions as single global constructs [13–16]. Such a reductionist approach may obscure substantial heterogeneity in symptom presentations. The severity of these conditions is typically assessed through standardized measures comprising observable symptoms [15], yet individuals with comparable total scores may experience distinctly different symptom patterns and clinical trajectories. This heterogeneity in symptom manifestations highlights the limitations of treating psychological and behavioral symptoms as uniform constructs [17]. In contrast, person-centered approaches, such as latent profile analysis, enable the identification of distinct subgroups characterized by different patterns and severity levels of specific Internet addiction and depressive symptoms, potentially revealing high-risk populations requiring immediate clinical attention and facilitating more targeted therapeutic interventions [18].

Furthermore, various sociodemographic characteristics and lifestyle factors are known to influence the manifestation of Internet addiction and depressive symptoms. Gender differences have been reported, with females potentially demonstrating elevated vulnerability to depressive symptoms [19], while males may exhibit higher susceptibility to Internet addiction [20]. Academic factors, including the degree program type (associate vs., bachelor's), disciplinary focus, and grade level progression, correlate with varying stress exposure and career trajectory clarity [13, 21]. Within China's specific context, ethnic minority status can introduce adaptation challenges related to acculturation and potential marginalization [22], while only-child status may influence family dynamics and psychological development [23]. Socioeconomic status, particularly household income, could function as a buffer against psychological distress by enhancing resource accessibility and mitigating financial stress [24]. Additionally, lifestyle choices are significant factors; regular physical activity is recognized for its mental health benefits [25], whereas excessive daily internet use duration is intrinsically linked to Internet addiction and its negative consequences [2]. Acknowledging these diverse influences provides essential foundations for identifying high-risk subgroups requiring enhanced surveillance and intervention.

Given that high-risk populations often experience more severe functional impairment and face elevated risks of adverse outcomes (e.g., academic failure, social withdrawal, and compromised quality of life), understanding the symptom dynamic within these vulnerable groups facilitates the identification of influential symptoms and their interconnections that contribute to sustained psychological distress, thereby enabling precise preventive and therapeutic interventions [26]. Network analysis theory conceptualizes psychiatric disorders as emergent phenomena arising from dynamic systems of interacting and mutually reinforcing symptoms [26]. This approach diverges from traditional methodologies that treat symptoms as interchangeable indicators of underlying disorders; instead, network analysis illuminates the complex ways in which specific symptoms activate and maintain others, thereby providing crucial insights into the mechanisms of psychopathology [26]. This analytical framework allows for the detection of central symptoms—those exerting the strongest influence within the symptom network—which may serve as optimal targets for clinical intervention [27]. Network analyses of Internet addiction and depressive symptoms have been conducted in several clinical populations [28–30]; however, the symptom interplay between these conditions remains unexplored among university students—a population warranting particular attention during their critical life transition period marked by considerable future uncertainties. In addition, previous studies suggest that individuals with elevated psychological distress often face increased suicide risk [10, 31, 32]; flow network analysis can further elucidate the specific symptomatic pathways through which Internet addiction and depressive symptoms cascade into suicidal thoughts and behaviors, thereby identifying precise targets for suicide prevention among these vulnerable populations [33].

Integrating the person-centered and network-based approaches, this study aimed to elucidate the co-occurrence patterns and symptom interactions of Internet addiction and depressive symptoms among Chinese university students. Specifically, we sought to identify distinct profiles based on Internet addiction and depressive symptoms using latent profile analysis and subsequently examine sociodemographic and lifestyle predictors of profile membership. Building upon the person-centered stratification, we further aimed to uncover the interconnected symptom networks, identify central and bridge symptoms, among high-risk populations and to explore the symptomatic pathways leading to suicide risk. This multidimensional analytical framework contributes to understanding the heterogeneous presentations of psychological distress among university students and provides empirical evidence for targeted intervention strategies.

## 2. Methods

### 2.1. Study Design and Participants

This study utilized a multicenter cross-sectional design, adhering to the Strengthening the Reporting of Observational Studies in Epidemiology (STROBE) statement for cross-sectional studies [34]. Two universities were selected from each of the seven major geographical regions of Mainland China (north, northeast, east, south, central, southwest, and northwest), resulting in the participation of 14 institutions. The seven regions correspond to the standard major geographical divisions of Mainland China. Selecting two universities per region was intended to balance breadth of geographical coverage with recruitment feasibility. Eligible participants were required to meet the following criteria: (1) university students aged 16 or above; (2) the ability to comprehend and complete the questionnaire; and (3) willingness to provide informed consent for participation.

### 2.2. Data Collection

Data collection was conducted from April to July 2024 using a convenience sampling method. The research process began with the principal investigator obtaining consent from university administrators at each participating institution, who then facilitated participant recruitment by disseminating an e-flyer containing a WeChat QR code to potential students within their respective institutions. Students who scanned this code were directed to the questionnaire hosted on the survey star platform (www.wjx.cn). Before accessing the survey, participants were presented with a concise overview of the study and assurances of anonymity and confidentiality. Informed consent was obtained electronically, with participants required to click the “agree” button before proceeding with the questions. Participants could decline participation by selecting “exit” at any point. To ensure data quality and prevent duplicate submissions, the platform was programed to accept single submissions per IP address and implemented a paginated format requiring full completion of each section.

### 2.3. Measures

#### 2.3.1. Sociodemographic and Lifestyle Characteristics

Participants' characteristics were collected using a self-administered questionnaire. Sociodemographic variables included age, gender, education level, major, grade, only child status, ethnicity, residence, and monthly household income. Lifestyle characteristics encompassed daily physical activity and daily internet use.

#### 2.3.2. Internet Addiction Symptoms

Internet addiction symptoms were evaluated using the 6-item Internet Addiction Test (IAT-6) [35], a brief version of the original IAT [3], designed to measure characteristics and behaviors related to Internet addiction (e.g., compulsivity, escapism, and dependency) during the past month. Each item is rated on a 5-point Likert scale ranging from 1 (“rarely”) to 5 (“always”). Total scores are obtained by summing all item scores (range: 6–30), with higher scores indicating greater Internet addiction severity [35]. A representative item asks: “Do you feel depressed, moody, or nervous when you are offline, which goes away once you are back online?” Previous studies have confirmed the scale's good psychometric properties in Chinese university students [35, 36]. In our study, the 6-item IAT exhibited excellent internal consistency, with a Cronbach's *α* of 0.899 and a Guttman split-half reliability of 0.898.

#### 2.3.3. Depressive Symptoms

Depressive symptoms were measured using the 9-item Patient Health Questionnaire (PHQ-9) [37]. The questionnaire evaluates the frequency of depressive symptoms over the past two weeks, with items rated on a 4-point Likert scale from 0 (“not at all”) to 3 (“almost every day”). Total scores range between 0 and 27, with higher scores indicating more severe depressive symptoms [38]. An example item is: “Feeling tired or having little energy”. Previous validation studies have confirmed the acceptable psychometric properties of the Chinese version of the PHQ-9 [39]. The current study found high reliability, with a Cronbach's alpha of 0.934 and a Guttman's split-half reliability of 0.868.

#### 2.3.4. Suicide Risk

Suicide risk was assessed using three standard suicide-related items from the Youth Risk Behavior Survey, examining suicidal ideation, suicidal plan, and suicidal attempt during the past 12 months [40]. Suicidal ideation and suicidal plan were each determined by one question “Did you ever seriously consider attempting suicide?”, and “Did you make a plan about how you would attempt suicide?”, respectively, with “Yes” (1) and “No” (0) response options. Suicide attempt was determined by the question “How many times did you actually attempt suicide?”. Responses to this item were dichotomized into “No attempts” (0) or “One or more attempts” (1). Participants were classified as having suicide risk if they responded positively to any of these three items.

### 2.4. Statistical Analysis

Statistical analyses were performed using R version 4.3.2. Descriptive statistics were computed for all variables; categorical variables were presented as frequencies and percentages, while continuous variables were expressed as means ± standard deviations (SD). All tests were two-tailed, with statistical significance set at *p* < 0.05.

#### 2.4.1. Latent Profile Analysis

To identify distinct patterns of co-occurring Internet addiction and depressive symptoms, we implemented the latent profile analysis based on the item scores of IAT-6 and PHQ-9 using the R package “tidyLPA”. Models containing one to six latent profiles were estimated, and for each model, we calculated the Akaike Information Criterion (AIC), Bayesian Information Criterion (BIC), sample-size adjusted BIC (aBIC), entropy, and bootstrap likelihood ratio test (BLRT) [41]. Model selection was guided by established criteria, prioritizing models with (1) lower relative fit information criteria, including lower AIC, BIC, and aBIC; (2) high entropy of at least 0.8; and (3) a significant BLRT *p*-value, indicating an improved model fit compared to the *k*-1 class solution [42]. Upon profile identification, between-profile differences in sociodemographic and lifestyle characteristics were assessed using chi-square tests or ANOVA, as appropriate. To characterize profile membership determinants, we performed multivariate logistic regression to examine sociodemographic predictors.

#### 2.4.2. Network Analysis

To further explore the symptom interactions and identify central symptoms within the identified high-risk populations, we conducted network analysis on the subgroup showing the highest co-occurrence of Internet addiction and depressive symptoms. The network was constructed using regularized partial correlation analyses with LASSO regularization, optimized via the Extended Bayesian Information Criterion (EBIC), using the default hyperparameter gamma (*γ*) = 0.5 to balance model sparsity and fit [43, 44]. The network structure was visualized using the Fruchterman–Reingold algorithm with a “spring” layout, positioning more influential nodes centrally and closely connected nodes proximally. Network estimation and visualization were implemented through the R packages “qgraph” and “bootnet”. In the resultant network, nodes represented individual symptoms, and edges denoted the unique associations between symptoms after controlling for other nodes [33]. Edge thickness reflected the strength of association, with solid and dashed lines indicating positive and negative correlations, respectively [33].

To quantify the relative importance of symptoms within the network, we calculated the expected influence (EI), which appropriately handles networks containing both positive and negative edges, thereby better reflecting the potential flow of activation through the overall network structure [43]. Furthermore, bridge symptoms connecting Internet addiction and depressive symptom clusters were identified using bridge EI, implemented through the “*networktools*” R package [45, 46]. This metric quantifies the extent to which a symptom in one community is expected to influence symptoms in the other community [47]. Network robustness was evaluated through edge accuracy assessment and centrality stability analysis. Edge accuracy was assessed via nonparametric bootstrapping with 1000 bootstrap samples. Centrality stability was examined using a case-dropping bootstrap approach. The correlation stability coefficient (CS-C) quantified the network stability, with values exceeding 0.5 considered satisfactory [48]. Intranetwork comparisons for all edge weights, node EI, and bridge EI indices were conducted using bootstrap confidence intervals (CIs), with differences considered statistically significant when the CIs excluded zero. Subsequently, to elucidate the potential progression toward suicide risk in the high-risk subgroup, we constructed a flow diagram illustrating the symptomatic pathways connecting Internet addiction, depressive symptoms, and suicidal risk.

## 3. Results

### 3.1. Study Sample and Descriptive Statistics

Our online survey garnered 32,537 submissions; 1545 submissions were excluded during data cleaning due to invalid response patterns (e.g., indications of random or inconsistent responding) or the presence of extreme outliers. The final sample consisted of 30,992 valid responses, yielding an effective response rate of 95.25%.

Table S1 summarizes the participants' sociodemographic and lifestyle characteristics. The sample comprised predominantly female students (60.6%), with a mean age of 19.31 ± 1.31 years. First-year students constituted the majority (52.3%), with most pursuing associate degrees (53.4%) across liberal arts (38.3%), science and engineering (39.0%), and medical (22.7%) disciplines. Most participants were not-only children (71.8%) from families with monthly incomes below 5000 RMB (72.3%). Approximately 70% of the participants reported above 4 h of daily internet use, while only 27.6% engaged in physical activity exceeding 1 h daily. Additionally, the mean scores for Internet addiction and depressive symptoms were 10.55 ± 4.99 and 4.34 ± 5.07, respectively, with 8.4% of participants reporting suicide risk.

### 3.2. Potential Latent Profiles Identified by Latent Profile Analysis

Latent profile analysis models with one to six profiles were examined (Figure S1). The three-profile solution was selected as optimal based on a comprehensive evaluation balancing statistical fit indices with theoretical interpretability and parsimony (Table 1). Although information criteria (AIC, BIC, and aBIC) slightly decreased beyond three profiles—a common occurrence in large samples—multiple key indicators favored the three-profile model. This solution achieved the highest entropy value (0.958), indicating superior classification certainty, and a significant BLRT (*p* < 0.01). Crucially, the three profiles were distinct, clinically interpretable, and represented meaningful subgroups. In contrast, solutions with four or more profiles showed declining entropy and yielded smaller, less distinct subgroups lacking clear conceptual underpinning. For instance, the additional profile in the 4-profile solution appeared to be a variation of an existing profile rather than a qualitatively unique pattern. This suggested that increasing model complexity beyond three profiles did not capture genuinely different configurations and risked overfitting. Furthermore, the clinical relevance and validity of the three profiles were supported by significant differences in suicide risk across the profiles (*χ*^2^ = 3994.416, *p* < 0.001), with the comorbidity profile exhibiting markedly elevated risk (42.7%) compared to the at-risk (13.7%) and Healthy (2.1%) profiles (Table S1).

**Figure 1:**
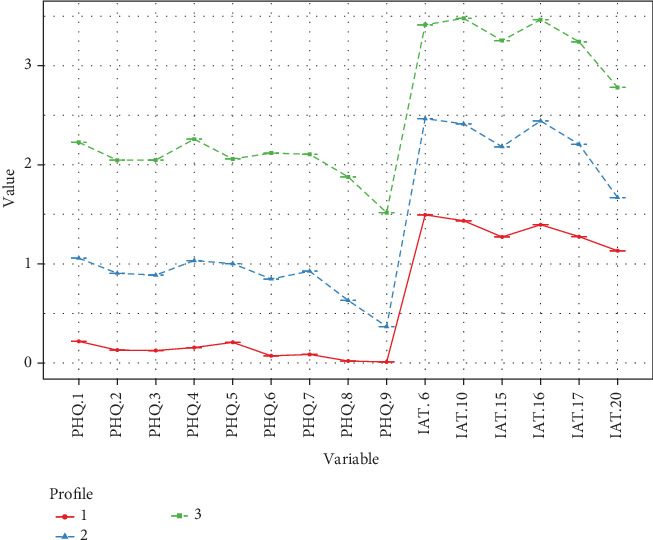
Symptom severity scores across the three latent profiles (*N* = 30,992). Note: *X*-axis represents Internet addiction (IAT-6) and depression (PHQ-9) items; *Y*-axis represents mean item scores.

Figure 1 illustrates the distinct symptom patterns across the three profiles. The profiles were characterized as follows: Profile 1 (“Healthy profile”, *n* = 18,380, 59.31%) exhibited minimal symptoms of both Internet addiction (8.00 ± 2.96) and depression (1.04 ± 1.52), indicating adaptive Internet use and psychological well-being. Profile 2 (“At-risk profile”, *n* = 10,866, 35.06%) showed elevated Internet addiction symptoms (13.40 ± 4.28) coupled with mild depressive symptoms (7.69 ± 2.36), suggesting potential vulnerability. Profile 3 (“Comorbidity profile”, *n* = 1746, 5.63%) demonstrated pronounced symptoms of both Internet addiction (19.64 ± 5.83) and depression (18.27 ± 4.35), reflecting significant comorbidity of problematic Internet use and depressive symptoms.

### 3.3. Sociodemographic and Lifestyle Factors Influencing Profile Membership

**Figure 2:**
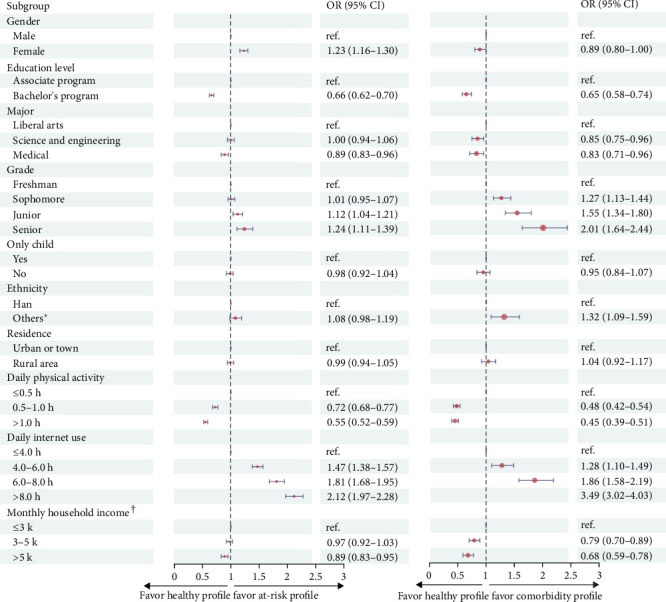
Multivariate logistic regression analyses of sociodemographic and lifestyle factors influencing profile membership. Note: OR, odds ratio; CI, confidence interval. *⁣*^*∗*^ “Others” in ethnicity category refers to ethnic minorities in China (non-Han ethnic groups). ^†^ Monthly household income in CNY (3k ≈ 410 USD, 5k ≈ 680 USD).

Chi-square tests and ANOVA revealed significant between-profile differences across all participant characteristics (all *p* < 0.05). Subsequent multivariate logistic regression analyses, using the Healthy profile as reference, revealed distinct patterns of correlates across profiles regarding students' background characteristics and behaviors (Figure 2). Specifically, female students showed higher odds of at-risk profile membership (OR = 1.23, 95% CI: 1.16–1.30), while ethnic minority students exhibited elevated odds of comorbidity profile membership (OR = 1.32, 95% CI: 1.09–1.59). Students in bachelor's programs, compared to associate programs, demonstrated consistently lower odds of both At-risk (OR = 0.66, 95% CI: 0.62–0.70) and comorbidity profile membership (OR = 0.65, 95% CI: 0.58–0.74). Compared to liberal arts majors, students in science-related and medical fields showed lower odds of comorbidity profile membership (OR = 0.85, 95% CI: 0.75–0.96; OR = 0.83, 95% CI: 0.71–0.96, respectively). Higher grade levels, relative to freshmen, were associated with incrementally increased risk, particularly among seniors (At-risk: OR = 1.24, 95% CI: 1.11–1.39; Comorbidity: OR = 2.01, 95% CI: 1.64–2.44). Higher household income (>5 k CNY) showed protective effects across both analyses (At-risk: OR = 0.89, 95% CI: 0.83–0.95; Comorbidity: OR = 0.68; and 95% CI: 0.59–0.78).

A clear gradient effect was observed in lifestyle-related factors. Compared to those reporting ≤0.5 h daily physical activity, students engaging in longer durations showed progressively lower odds of problematic profiles, with >1.0 h daily exercise demonstrating the strongest protective effect (At-risk: OR = 0.55, 95% CI: 0.52–0.59; Comorbidity: OR = 0.45, 95% CI: 0.39–0.51). Conversely, relative to those reporting ≤4.0 h daily Internet use, increased duration showed stepwise elevation in odds of problematic profiles, with the strongest association observed for >8.0 h daily use (At-risk: OR = 2.12, 95% CI: 1.97–2.28; Comorbidity: OR = 3.49, 95% CI: 3.02–4.03).

### 3.4. Network Structure, Central Symptoms, and Bridge Symptoms in the Comorbidity Profile

**Figure 3:**
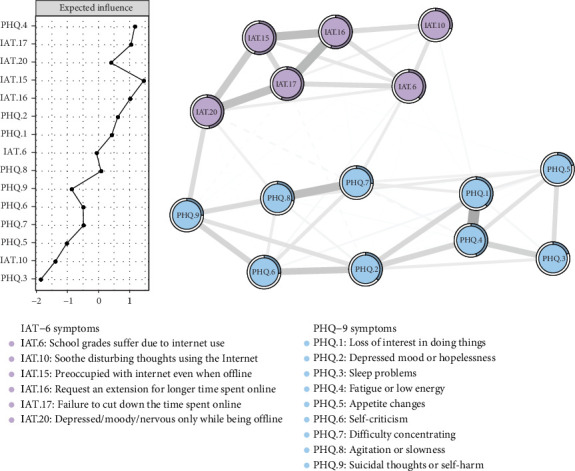
Network analysis of Internet addiction and depressive symptoms in the comorbidity profile.

Figure 3 illustrates the network structure of Internet addiction and depressive symptoms among individuals classified into the comorbidity profile through latent profile analysis. The network encompassed 15 nodes and 79 nonzero edges out of a possible 105 connections. The network topology revealed a relatively dense structure, with 75.24% of possible edges present, although the average strength of these connections was weak to mild (mean edge weight = 0.066). Central symptom analysis using the EI index (Table S2) identified IAT.15 as the most influential node (“feeling preoccupied with the Internet when off-line”; EI = 1.450), followed by PHQ.4 (“feeling tired or having little energy”; EI = 1.165). Furthermore, bridge EI analysis (Table S2) identified IAT.20 (“feeling depressed/moody/nervous when offline”; bridge EI = 0.205), IAT.6 (“school grades suffer”; bridge EI = 0.165), IAT.10 (“soothe disturbing thoughts using the Internet”; bridge EI = 0.148), and PHQ.7 (“difficulty concentrating”; bridge EI = 0.144) as the most influential bridge symptoms connecting the Internet addiction and depression clusters. The network structure demonstrated high accuracy, as evidenced by narrow 95% CIs in the bootstrap analysis of edge weights (Figure S2). The stability analysis yielded robust EI and bridge EI centrality estimates, with CS coefficients exceeding 0.75 [48] (Figure S3). Differential analysis through bootstrap tests revealed significant heterogeneity in edge weights (Figures S4) and centrality indices (Figures S5–S6), confirming the significantly higher centrality of IAT.15 and PHQ.4 relative to other symptoms, and highlighting the distinct roles of key bridge symptoms.

### 3.5. Pathways to Suicide Risk in the Comorbidity Profile

**Figure 4:**
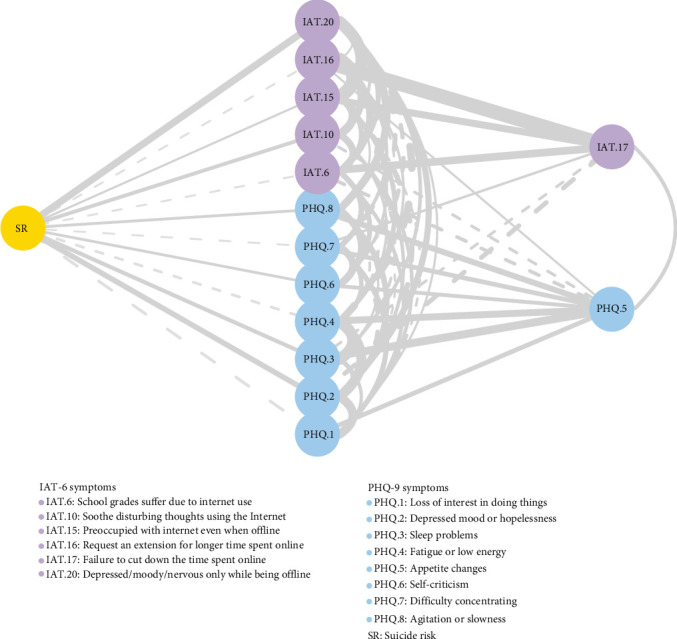
The flow diagram of network showing how Internet addiction and depressive symptoms connect to suicide risk in the comorbidity profile. Note: Symptoms positioned in the middle of the diagram indicate direct connections to suicide risk, while those on the right represent indirect connections. PHQ.9 (“Thoughts that you would be better off dead or of hurting yourself in some way”) was excluded from the analysis due to substantial conceptual overlap with the suicide risk outcome and identified item redundancy.

The flow diagram (Figure 4) mapping the symptomatic pathways connecting Internet addiction, depressive symptoms, and suicide risk revealed both direct and indirect connections to suicide risk in the comorbidity profile. Across all examined pathways, the strongest associations with suicide risk were identified for withdrawal manifestations within Internet addiction symptoms (“feeling depressed, moody, or nervous when offline”) (IAT.20; edge weight = 0.150) and depressed mood within depressive symptoms (“feeling down, depressed, or hopeless”) (PHQ.2; edge weight = 0.108).

## 4. Discussion

This large-scale study represents the first comprehensive investigation integrating person-centered and network approaches to delineate the heterogeneous presentations and symptom interactions of Internet addiction and depression among Chinese university students. Our findings revealed three distinct symptom profiles: a healthy profile (59.31%), an at-risk profile (35.06%), and a comorbidity profile (5.63%). Significant between-profile differences emerged across sociodemographic and lifestyle characteristics, with female gender, ethnic minority status, higher grade levels, and prolonged Internet use associated with elevated risk of problematic profile, while bachelor's degrees, medical majors, higher household income, and regular physical activity conferred protective effects. Moreover, the network analysis of the high-risk comorbidity profile identified Internet preoccupation (IAT.15) and fatigue/low energy (PHQ.4) as central symptoms, highlighted specific bridge symptoms (e.g., IAT.20, PHQ.7) linking the two conditions, and revealed withdrawal manifestations (IAT.20) and depressed mood (PHQ.2) as the strongest direct connections to suicide risk. These findings advance our understanding of risk stratification and symptom interactions in comorbid Internet addiction and depression, providing evidence-based targets for early identification and intervention among university students.

Our latent profile analysis unveiled three distinct patterns of Internet addiction and depressive symptom co-occurrence among Chinese university students, highlighting the heterogeneous nature of psychological distress in this population. The predominant Healthy profile (59.31%) exhibited minimal symptoms, indicating adaptive Internet use and psychological well-being, while the at-risk profile (35.06%) showed elevated Internet addiction with mild depressive symptoms, suggesting subclinical psychological disturbances. The comorbidity profile (5.63%), though smallest in proportion, represented a vulnerable subgroup demonstrating pronounced symptoms of both conditions. This consistent co-occurrence pattern aligns with previous evidence of bidirectional relationships between Internet addiction and depression [15, 49], potentially driven by shared neurobiological mechanisms including altered reward processing and emotional regulation difficulties [50, 51]. The synchronized severity progression across profiles reflects a theoretical framework where excessive Internet use and depressive symptoms mutually reinforce each other through maladaptive coping cycles, particularly manifested in the comorbidity profile's severe symptomatology.

The examination of sociodemographic and lifestyle correlates revealed distinct risk and protective factors associated with profile membership. Analyses of sociodemographic characteristics indicated that female students showed higher odds of at-risk profile membership while ethnic minority students exhibited increased likelihood of comorbidity profile membership, suggesting heightened psychological vulnerability due to gender-specific stress responses and sociocultural adaptation challenges [19, 22]. Enrollment in bachelor's programs demonstrated protective effects against both problematic profiles compared to associate degree programs, reflecting advantages in academic resources, learning environment, and career prospects within the Chinese educational context. Students majoring in science and medicine exhibited lower odds of comorbidity profile membership relative to those in liberal arts, possibly due to more favorable career opportunities and academic advancement prospects [21]. Higher grade levels showed incrementally increased risks, with seniors demonstrating the highest vulnerability, suggesting cumulative effects of academic pressure and transitional challenges. Furthermore, higher household income exhibited protective effects across both At-risk and comorbidity profiles, reflecting how socioeconomic resources may buffer against psychological distress through enhanced access to support and resources [24]. Lifestyle factors demonstrated clear dose–response relationships: increased physical activity duration showed progressively stronger protective effects, likely through improved stress management and emotional regulation mechanisms [25], while longer daily Internet use was associated with stepwise elevation in risk for both problematic profiles, potentially by compromising healthy coping behaviors and daily functioning [6]. These findings provide empirical support for risk stratification and highlight the potential for developing targeted screening programs or tailored prevention strategies within university settings. Specifically, focusing screening efforts on identified vulnerable groups, such as ethnic minorities and senior students, while promoting protective factors like physical activity and balanced Internet use through campus-wide initiatives, could inform effective public health policies.

Network analyses of the comorbidity profile identified Internet preoccupation (IAT.15) from Internet addiction symptoms and fatigue/low energy (PHQ.4) from depressive symptoms as the most central nodes in the network structure [27]. Theoretically, the high centrality of Internet preoccupation (IAT.15) aligns strongly with cognitive–behavioral models of Internet addiction, such as the I-PACE model [52], which posits that persistent, intrusive thoughts about Internet use are core cognitive components that drive and maintain addictive behaviors by occupying cognitive resources and triggering craving. The central position of fatigue/low energy (PHQ.4), a cardinal symptom of depression [37], underscores its systemic impact, potentially reflecting underlying neurobiological alterations or serving as a key driver of behavioral inactivity, thereby maintaining the depressive state [27]. Practically, the identification of these central symptoms offers crucial therapeutic implications, suggesting that interventions, such as mindfulness-based techniques targeting persistent preoccupation (IAT.15) or behavioral activation strategies addressing fatigue/low energy (PHQ.4) may yield cascading positive effects throughout the symptom network, thereby optimizing treatment efficiency.

Additionally, our bridge symptom analysis illuminated specific insights into the symptoms linking the Internet addiction and depression clusters within the comorbidity profile. Notably, negative affect when offline (IAT.20), academic impairment (IAT.6), using Internet for emotional regulation (IAT.10), and concentration difficulties (PHQ.7) emerged as prominent bridge symptoms. The pronounced bridging effect of IAT.20 suggests that offline emotional dysregulation may act as a critical conduit, potentially activating or exacerbating depressive states. Similarly, Internet-based emotional coping (IAT.10) and functional deterioration (IAT.6) represent behavioral and consequential bridges linking mood disturbances to problematic Internet use patterns. From the depression cluster, concentration difficulties (PHQ.7) exhibited the strongest bridging effect, possibly reflecting how cognitive impairments associated with depression compromise offline functioning and foster increased digital engagement. These findings corroborate theoretical models positing reciprocal symptom reinforcement in comorbid presentations [13, 16, 47] and suggest that interventions targeting these specific bridge symptoms—such as enhancing offline coping strategies, providing academic support, or offering cognitive training to improve attentional control—may effectively disrupt the self-perpetuating cycle between Internet addiction and depression in vulnerable students.

Further examination of the pathways to suicide risk revealed two robust routes of direct influence. Internet withdrawal symptoms (IAT.20), manifested as feeling depressed, moody, or nervous when offline, demonstrated the strongest direct connection to suicide risk, suggesting that emotional dysregulation during offline periods may serve as a critical vulnerability factor [53]. Concurrently, depressed and hopeless mood (PHQ.2) emerged as another robust predictor of suicide risk, potentially operating through its impact on negative self-evaluation and hopelessness [54]. These findings delineate the specific pathways through which Internet addiction and depressive symptoms contribute to suicide risk, highlighting that suicide prevention strategies should precisely target both Internet withdrawal symptoms and depressive mood to achieve optimal therapeutic outcomes in this vulnerable subgroup.

This study innovatively integrates person-centered and network-based approaches in Internet addiction and depression research among university students to uncover distinct symptom profiles, identify central and bridge symptoms, and delineate specific pathways to suicide risk. However, several limitations of this study warrant consideration. First, the cross-sectional design precludes causal inferences regarding the temporal relationships between Internet addiction and depressive symptoms. While our cross-sectional latent profile analysis identified high-risk subgroups, longitudinal studies, such as Zhang et al. [55] have demonstrated that symptom trajectories (e.g., escalating comorbidity vs., remission) may exhibit distinct etiological pathways, suggesting the need for temporal analyses to validate profile stability and capture developmental transitions between profiles. Second, despite implementing quality control measures, our findings relied solely on self-report measures, which may be subject to recall bias and social desirability, particularly in reporting sensitive issues, such as suicidal behaviors. Future research could benefit from incorporating multiple assessment methods, such as clinical interviews, ecological momentary assessment, and passive sensing data to triangulate symptom manifestations. Third, our study utilized convenience sampling, introducing potential selection bias inherent to nonrandom sampling. Although our multicenter strategy aimed to enhance sample diversity by including institutions from all seven major geographical regions of Mainland China, the convenience nature of the sampling means we cannot guarantee perfect regional representativeness; future studies employing stratified random sampling are needed to confirm our findings in more representative samples. Finally, although we examined sociodemographic and lifestyle-related predictors of profile membership, other potentially important factors, such as academic stress, family relationships, and social support were not included in the current analysis, which may provide additional insights into the determinants of symptom patterns.

## 5. Conclusion

In conclusion, this study identified three distinct symptom profiles of Internet addiction and depression among Chinese university students and revealed specific sociodemographic and lifestyle factors predicting profile membership. The network analysis further highlighted Internet preoccupation and fatigue/low energy as central symptoms maintaining symptom interactions, while identifying key bridge symptoms—notably offline negative affect, Internet use for emotional regulation, academic impairment, and concentration difficulties—that facilitate symptom transmission between disorders. Additionally, Internet withdrawal symptoms and depressed mood emerged as critical pathways to suicide risk in the high-risk comorbidity profile. The identification of distinct profiles enables more targeted screening and intervention strategies, particularly for vulnerable populations, such as female students, ethnic minorities, and those with higher grade levels. Moreover, modifiable factors, such as regular physical activity and controlled Internet use time provide actionable insights for prevention programs. Notably, within the high-risk comorbidity profile, targeting the central symptoms of Internet preoccupation and fatigue may yield cascading benefits across the symptom network, while interventions addressing bridge symptoms could potentially disrupt the mutual reinforcement between Internet addiction and depression, and monitoring withdrawal symptoms and depressed mood could facilitate early identification of suicide risk. Future prevention and intervention strategies may incorporate these empirical findings to develop more precise and effective approaches for university students.

## Figures and Tables

**Table 1:** Latent profile model fit indices (*N* = 30,992).

Model	Log-like	AIC	BIC	aBIC	Entropy	BLRT (*p*)	Profile size (%)
1-profile	−557,695.1	1115,450.3	1115,700.5	111,5605.2	1.000	<0.01	1
2-profile	−472,052.8	944,197.7	944,581.4	944,435.2	0.944	<0.01	69.12/30.88
**3-profile**	**−435,794.2**	**871,712.5**	**872,229.7**	**872,032.6**	**0.958**	**<0.01**	**59.31/35.06/5.63**
4-profile	−420,933.8	842,023.5	842,674.2	842,426.3	0.947	<0.01	54.60/25.14/14.69/5.57
5-profile	−409,927.8	820,043.6	820,827.7	820,529.0	0.936	<0.01	47.98/21.00/18.31/8.65/4.06
6-profile	−401,098.7	802,417.4	803,335.0	802,985.4	0.936	<0.01	46.60/17.01/14.75/9.56/8.40/3.68

*Note:* Bold entries reflect the selected model.

Abbreviations: AIC, akaike information criterion; BIC, Bayesian information criterion; BLRT, bootstrap likelihood ratio test.

## Data Availability

The data of this study are available from the corresponding author upon reasonable request.

## Supporting Information

Supporting Information Table S1 presents sociodemographic and lifestyle characteristics of the participating students. Figure S1 illustrates the item-level symptom patterns for different latent profile solutions (1–6 profiles). Table S2 provides standardized values for nodes' Expected Influence and bridge Expected Influence. Figures S2–S6 present network analysis validation results, including bootstrapped confidence intervals of edge weights, network stability using case-drop procedure, and bootstrapped difference tests of edge weights and nodes' Expected Influence and bridge Expected Influence.
