# Supplementary material for: Internet Addiction and Depressive Symptoms in University Students: Latent Profiles, Network Structure, and Symptomatic Pathways to Suicide Risk
Source: Depress Anxiety. 2025 Jul 12;2025:4591408. doi: 10.1155/da/4591408 (PMC12276061; doi:10.1155/da/4591408)
Supplement: Supporting Information — Table S1 presents sociodemographic and lifestyle characteristics of the participating students. Figure S1 illustrates the item-level symptom patterns for different latent profile solutions (1–6 profiles). Table S2 provides standardized values for nodes' Expected Influence and bridge Expected Influence. Figures S2–S6 present network analysis validation results, including bootstrapped confidence intervals of edge weights, network stability using case-drop procedure, and bootstrapped difference tests of edge weights and nodes' Expected Influence and bridge Expected Influence. [file 4591408.f1.pdf]

Supplementary Materials

Content

Table S1. Sociodemographic and lifestyle characteristics of the participating students. 2

Figure S1. Item-level symptom patterns for different latent profile solutions (1-6 profiles). 4

Table S2. Standardized values for nodes' Expected Influence and bridge Expected Influence. 5

Figure S2. Bootstrapped confidence intervals of edge weights. 6

Figure S3. The stability of network using the case-drop procedure. 6

Figure S4. Bootstrapped difference test of edge weights...7

Figure S5. Bootstrapped difference test of the nodes' Expected Influence. 8

Figure S6. Bootstrapped difference test of the nodes' bridge Expected Influence. ..8

Table S1. Sociodemographic and lifestyle characteristics of the participating students (N = 30992).

**Characteristics**	Overall, N = 30992	Healthy group, n = 18380	At-risk group, n = 10866	Comorbidity group, n = 1746	F / χ^2	P-value
Age, yrs (Mean ± SD)	19.31 ± 1.31	19.25 ± 1.30	19.37 ± 1.30	19.53 ± 1.40	134.182	< 0.001
Gender, n (%)					95.628	< 0.001
Male	12220 (39.4)	7616 (41.4)	3883 (35.7)	721 (41.3)		
Female	18772 (60.6)	10764 (58.6)	6983 (64.3)	1025 (58.7)		
Education level, n (%)					488.598	< 0.001
Associate program	16537 (53.4)	8863 (48.2)	6552 (60.3)	1122 (64.3)		
Bachelor's program	14455 (46.6)	9517 (51.8)	4314 (39.7)	624 (35.7)		
Major, n (%)					110.319	< 0.001
Liberal arts	11865 (38.3)	6711 (36.5)	4398 (40.5)	756 (43.3)		
Science and engineering	12101 (39.0)	7165 (39.0)	4264 (39.2)	672 (38.5)		
Medical	7026 (22.7)	4504 (24.5)	2204 (20.3)	318 (18.2)		
Grade, n (%)					273.328	< 0.001
Freshman	16206 (52.3)	10001 (54.4)	5481 (50.4)	724 (41.5)		
Sophomore	8931 (28.8)	5366 (29.2)	3038 (28.0)	527 (30.2)		
Junior	4261 (13.7)	2241 (12.2)	1693 (15.6)	327 (18.7)		
Senior	1594 (5.1)	772 (4.2)	654 (6.0)	168 (9.6)		
Only child, n (%)					11.541	0.003
Yes	8747 (28.2)	5089 (27.7)	3112 (28.6)	546 (31.3)		
No	22245 (71.8)	13291 (72.3)	7754 (71.4)	1200 (68.7)		
Ethnicity, n (%)					9.417	0.009
Han	28436 (91.8)	16805 (91.4)	10040 (92.4)	1591 (91.1)		
Others*	2556 (8.2)	1575 (8.6)	826 (7.6)	155 (8.9)		
Residence, n (%)					13.535	0.001
Urban or town	15160 (48.9)	8836 (48.1)	5432 (50.0)	892 (51.1)		
Rural area	15832 (51.1)	9544 (51.9)	5434 (50.0)	854 (48.9)		
Monthly household income†, n (%)					44.817	< 0.001
≤ 3 k	10951 (35.3)	6327 (34.4)	3918 (36.1)	706 (40.4)		

**Characteristics**	Overall, $N = 30992$	Healthy group, $n = 18380$	At-risk group, $n = 10866$	Comorbidity group, $n = 1746$	F / χ^2	P-value
3 ~ 5 k	11469 (37.0)	6873 (37.4)	4024 (37.0)	572 (32.8)		
> 5 k	8572 (27.7)	5180 (28.2)	2924 (26.9)	468 (26.8)		
Daily physical activity, n (%)					695.305	< 0.001
≤ 0.5 h	9081 (29.3)	4349 (23.7)	3914 (36.0)	818 (46.8)		
0.5 ~ 1.0 h	13354 (43.1)	8170 (44.5)	4618 (42.5)	566 (32.4)		
> 1.0 h	8557 (27.6)	5861 (31.9)	2334 (21.5)	362 (20.7)		
Daily internet use, n (%)					586.117	< 0.001
≤ 4.0 h	9092 (29.3)	6389 (34.8)	2376 (21.9)	327 (18.7)		
4.0 ~ 6.0 h	9903 (32.0)	6011 (32.7)	3483 (32.1)	409 (23.4)		
6.0 ~ 8.0 h	5576 (18.0)	2985 (16.2)	2269 (20.9)	322 (18.4)		
> 8.0 h	6421 (20.7)	2995 (16.3)	2738 (25.2)	688 (39.4)		
Internet addiction symptoms (Mean ± SD)	10.55 ± 4.99	8.00 ± 2.96	13.40 ± 4.28	19.64 ± 5.83	13730.236	< 0.001
Depressive symptoms (Mean ± SD)	4.34 ± 5.07	1.04 ± 1.52	7.69 ± 2.36	18.27 ± 4.35	23242.626	< 0.001
Suicide risk					3994.416	< 0.001
Yes	2616 (8.4)	386 (2.1)	1484 (13.7)	746 (42.7)		
No	28376 (91.6)	17994 (97. 9)	9382 (86.3)	1000 (57.3)		

Note. SD, standard deviation.

* "Others" in ethnicity category refers to ethnic minorities in China (non-Han ethnic groups).

† Monthly household income in CNY (3k≈410 USD, 5k≈680 USD).

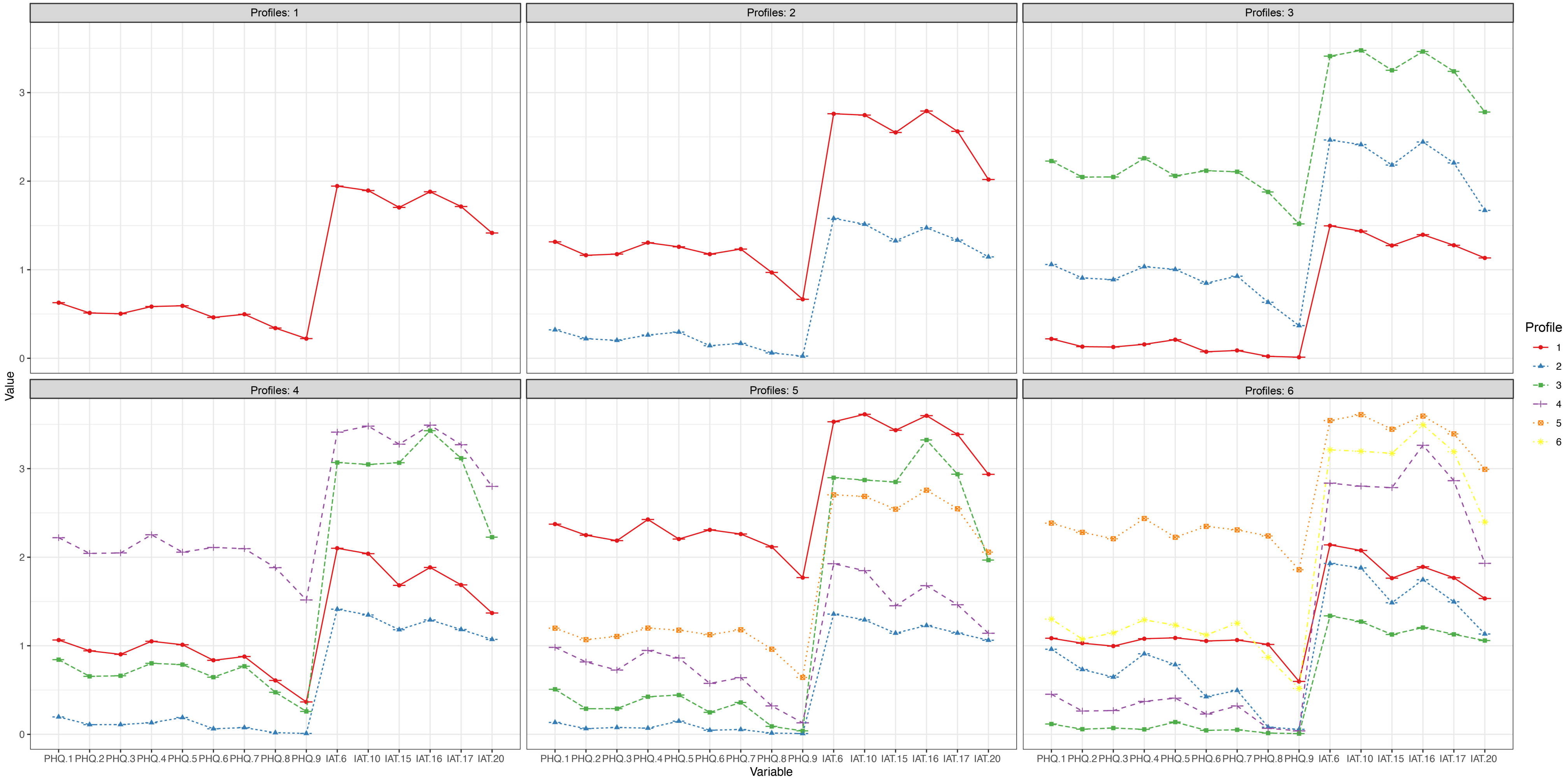

Figure S1. Item-level symptom patterns for different latent profile solutions (1-6 profiles).

Table S2. Standardized values for nodes' Expected Influence and bridge Expected Influence.

Nodes	Expected Influence values	bridge Expected Influence values
PHQ.1	0.423	0.081
PHQ.2	0.617	0.033
PHQ.3	-1.862	0.023
PHQ.4	**1.165**	0.066
PHQ.5	-1.025	0.043
PHQ.6	-0.494	0.102
PHQ.7	-0.484	**0.144**
PHQ.8	0.080	0.099
PHQ.9	-0.870	0.095
IAT.6	-0.065	**0.165**
IAT.10	-1.393	**0.148**
IAT.15	**1.450**	0.070
IAT.16	1.015	0.044
IAT.17	1.042	0.054
IAT.20	0.401	**0.205**

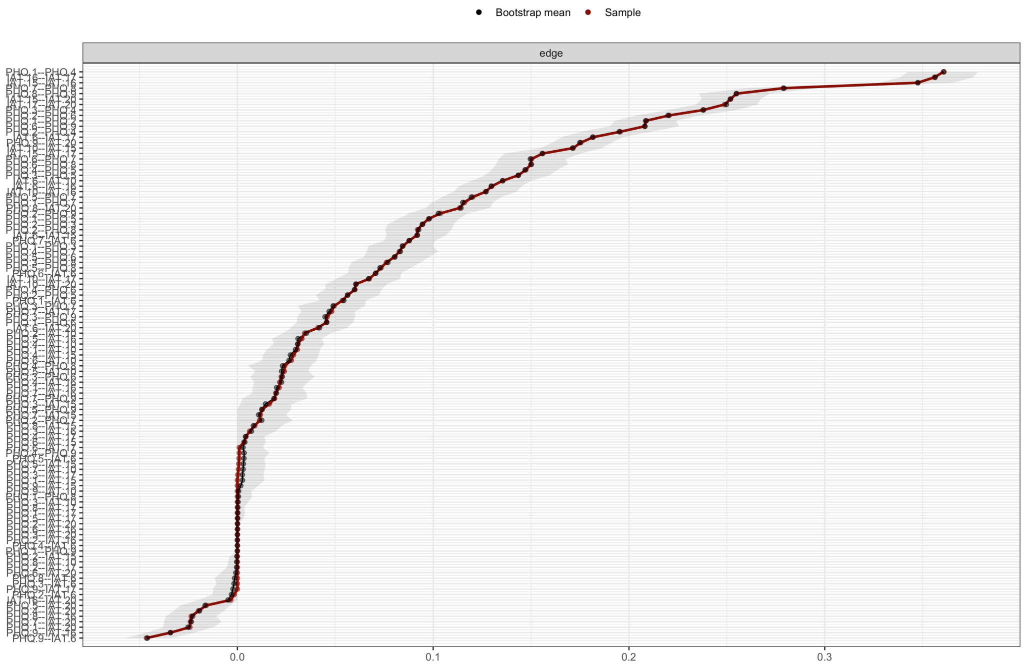

Figure S2. Bootstrapped confidence intervals of edge weights.

Note. The black dots indicate the values of each edge weight, ordered from the highest to the lowest value. The gray area represents the 95% Confidence Intervals of edge weights, estimated with the non-parametric bootstrap procedure (*bootnet* package). Wide intervals indicate lower stability and narrow intervals indicate higher stability.

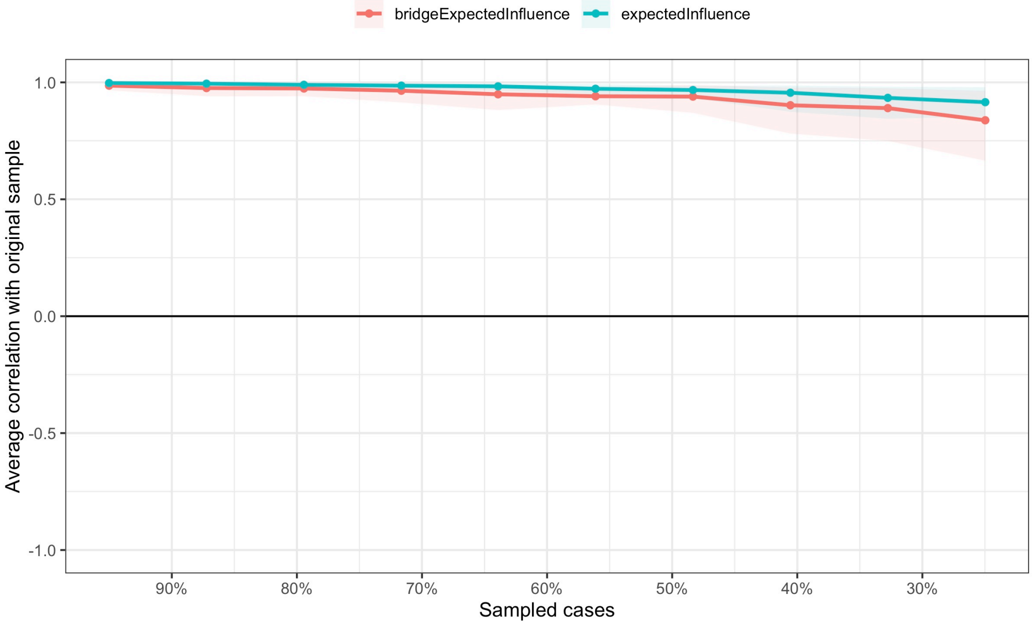

Figure S3. The stability of network using the case-drop procedure.

Note. CS-coefficient = 0.75

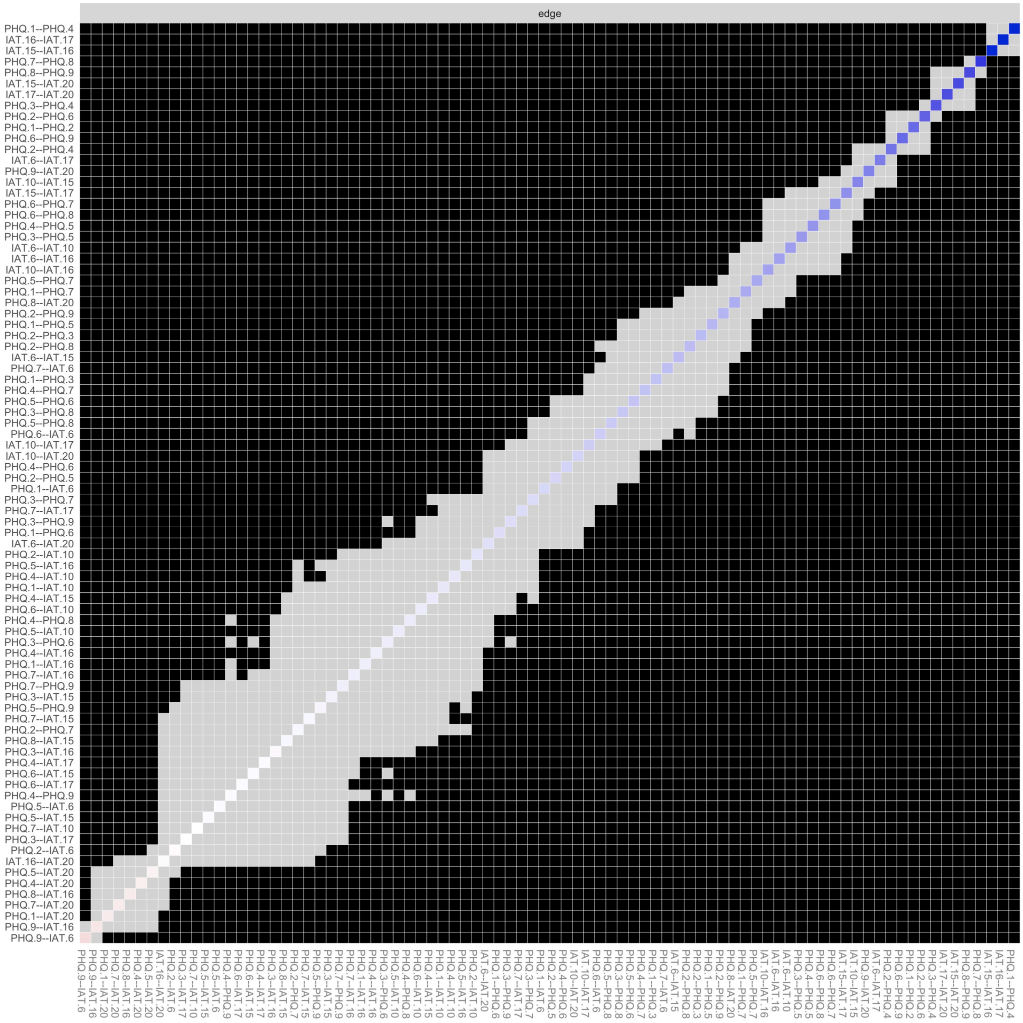

Figure S4. Bootstrapped difference test of edge weights.

Note. The y-axis and x-axis represent individual nodes within the psychological network. Colored boxes on the diagonal indicate the corresponding color of the edge within the network. Gray boxes indicate non-significant differences, while black boxes indicate significant differences.

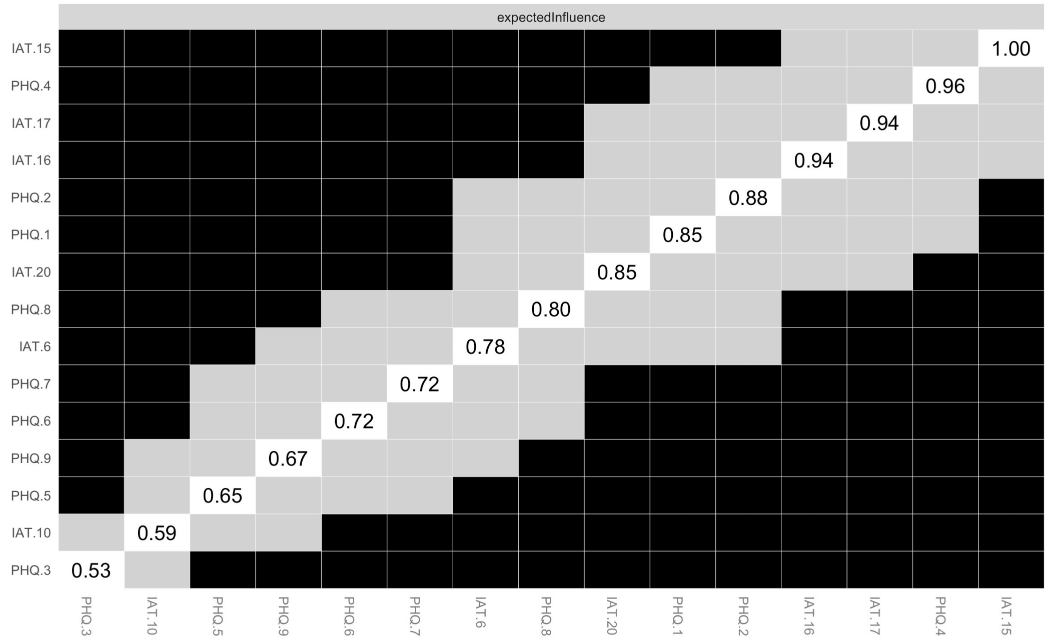

Figure S5. Bootstrapped difference test of the nodes' Expected Influence.

Note. The y-axis and x-axis represent individual nodes within the psychological network. Strength centrality values are plotted on the diagonal. Gray boxes indicate non-significant differences, while black boxes indicate significant differences.

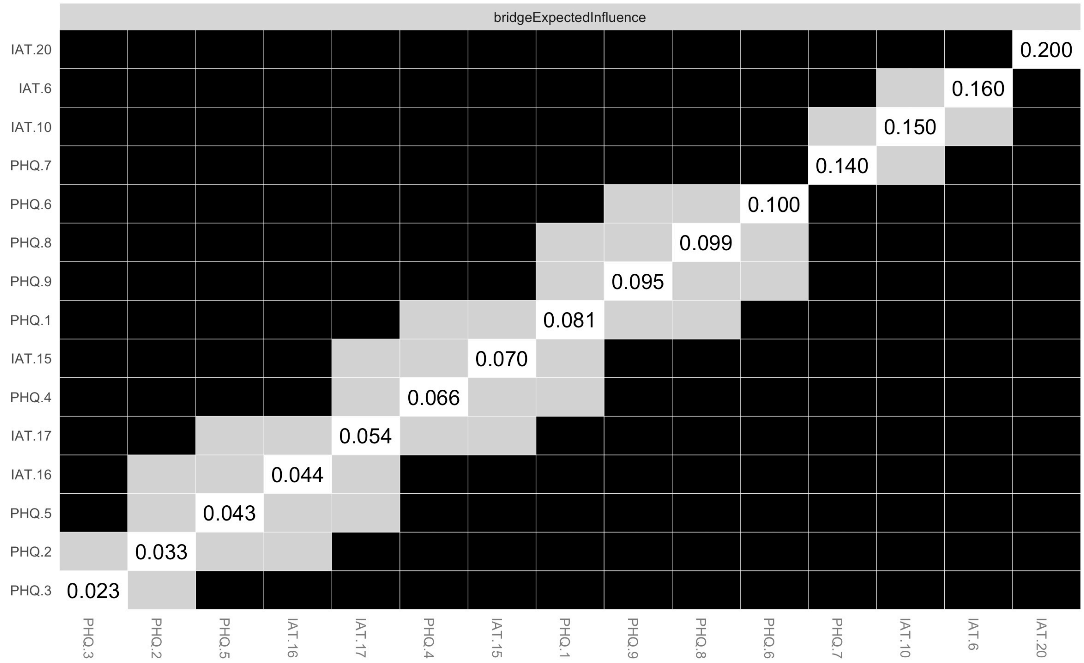

Figure S6. Bootstrapped difference test of the nodes' bridge Expected Influence.

Note. The y-axis and x-axis represent individual nodes within the psychological network. Strength centrality values are plotted on the diagonal. Gray boxes indicate non-significant differences, while black boxes indicate significant differences.
